# Cases of Cholera

**Published:** 1849-07

**Authors:** Clement A. Finley

**Affiliations:** U. S. Army; Newport Barracks, Newport, Ky.


*Cases of Cholera. Reported by* Clement A. Finley, M. D., U. S. Army.

Lobach, a musician, was found in his bunk in a state of collapse. He had been released from the guard-house the day previous, where he had been confined for drunkenness. In the evening of that day he was attacked with diarrhœa, which continued through the night, and till accidentally discovered by the hospital surgeon in the afternoon of the next day.

His state was as follows; No pulsation discernible in the radial artery; his surface cold, and covered with profuse perspiration; spasms of the muscles of the lower extremities: cold white tongue; serous discharges per anum et orem.

*Treatment.*—Large sinapisms over stomach and bowels; spinal irritation by applying a strip of flannel saturated with spts. of turpentine over the entire vertebral column, and passing a hot smoothing iron on it; this appeared to give immediate relief to the muscular spasms.

℞. Calomel xx., camph. gr. x., opium gr. j., given every hour till the arterial action was excited, then every two hours, till five doses had been taken; for some time I had hopes that he would recover, but, though the heart's action was for a time increased, and sustained by stimulants, carb. ammonia and brandy, he died by asthenia.

Recruit Rhett, a private, brought to the hospital after having diarrhœa about three days. Great debility; pulse in radial artery scarcely perceptible; husky voice; skin cold and clammy; tongue white and cold; serous discharges per anum et orem.

The treatment in this case was the same as in the preceding, except that opium was entirely omitted, and with the calomel and camphor, I twice gave sulph. quinine gr. x., and once five gr.

My whole attention was directed to sustain the heart's action, and prevent death by asthenia, till the nervous system should be restored to its tonic state, and with that the normal condition of the secretory organs.

The serous discharges continued at intervals of from three to five hours. After each discharge the calomel and camphor were exhibited for 24 hours, when the patient, for the first time, passed about half an ounce of high-colored urine; this secretion having been suppressed during the whole period of his treatment—and in an hour after a dejection, evidently produced by the calomel. From that time his recovery commenced; a slight ptyalism followed, which was probably owing to his having been salivated previously during his treatment for a severe attack of dysentery.

I was induced to drop the opium in the treatment of this case by the impression that it contributed to the atony of the nervous system, and counteracted the effects of the nervous tonics—quinine and camphor.

I have treated the disease in its incipient stage of diarrhœa, with the calomel and camphor alone, with uniform success.

I will mention that in the second case, spinal irritation was made as in the first, and there were no spasms or cramps during the attack.

In both cases creosote was given to check emesis, and with good effect. The quantity of camphor given with the calomel varied from two to ten grains, the calomel from ℥ss. to gr. x.; the evidence of improvement being the lengthened intervals between the discharges and the diminution of quantity. The thirst in both cases was excessive, for which ice and ice-water, to a limited extent, was allowed.

Newport Barracks, Newport, Ky., June 14, 1849.

